# Hydrogen prevents corneal endothelial damage in phacoemulsification cataract surgery

**DOI:** 10.1038/srep31190

**Published:** 2016-08-08

**Authors:** Tsutomu Igarashi, Ikuroh Ohsawa, Maika Kobayashi, Toru Igarashi, Hisaharu Suzuki, Masumi Iketani, Hiroshi Takahashi

**Affiliations:** 1 Department of Ophthalmology, Nippon Medical School 1-1-5 Sendagi, Bunkyo-ku, Tokyo 113-8602, Japan; 2 Biological Process of Aging, Tokyo Metropolitan Institute of Gerontology, 35-2 Sakae-cho, Itabashi-ku, Tokyo 173-0015 Japan; 3 Department of Pediatrics, Nippon Medical School 1-1-5 Sendagi, Bunkyo-ku, Tokyo 113-8602, Japan; 4 Department of Ophthalmology, Nippon Medical School Musashikosugi Hospital, 1-396 Kosugi-cho, Nakahara-ku, Kawasaki City, Kanagawa 211-8533, Japan

## Abstract

In phacoemulsification, ultrasound induces hydroxyl radical (·OH) formation, damaging corneal endothelium. Whether H_2_ can prevent such oxidative damage in phacoemulsification was examined by *in vitro* and *in vivo* studies. H_2_ was dissolved in a commercial irrigating solution. The effects of H_2_ against ·OH generation were first confirmed *in vitro* by electron-spin resonance (ESR) and hydroxyphenyl fluorescein (HPF). ESR showed a significantly decreased signal magnitude, and fluorescence intensity by oxidized HPF was significantly less in the H_2_-dissolved solution. The effects of H_2_ in phacoemulsification were evaluated in rabbits, comparing H_2_-dissolved and control solutions. Five hours after the procedure, the whole cornea was excised and subjected to image analysis for corneal edema, real-time semiquantitative PCR (qPCR) for heme oxygenase (HO)-1, catalase (CAT), superoxide dismutase 1 (SOD1), and SOD2 mRNA, and immunohistochemistry. Corneal edema was significantly less and the increases in anti-oxidative HO-1, CAT and SOD2 mRNA expressions were significantly suppressed in the H_2_ group. In addition, corneal endothelial cell expressions of two oxidative stress markers, 4-HNE and 8-OHdG, were significantly lower in the H_2_ group. In conclusion, H_2_ dissolved in the ocular irrigating solution protected corneal endothelial cells from phacoemulsification-induced oxidative stress and damage.

Most cataract surgeries are today performed by phacoemulsification, which uses high-intensity ultrasound (US) energy for fragmentation and emulsification of the lens. With the progress in surgical devices and the development of techniques, indications for phacoemulsification have now expanded to include mature cataract in which the lens nucleus is hard, so that greater US energy for emulsification is needed. Since human corneal endothelial cells lack mitotic activity *in vivo*, excessive damage to the tissue can cause significant decreases in endothelial cell density. Corneal endothelium maintains hydration of the corneal tissues by acting as a barrier and a draining pump against the aqueous humor; thus, decreases in endothelial cell density can induce irreversible corneal edema (bullous keratopathy; BK), in turn causing permanently blurred vision and pain. With an excessive amount of US energy^1^,^2^, collision of lens nucleus fragments with the corneal endothelium^3^, air bubbles^4^,^5^, or a localized temperature rise^6^,^7^,^8^ have been reported and are well known as factors damaging to the corneal endothelium. The development of free radicals has also been identified as a harmful factor associated with the use of US. Considering the oxidative insult to endothelial cells caused by free radicals, their presence in the anterior chamber may represent one of the most damaging factors during these procedures.

In the fields of physics and engineering, high-intensity US oscillating in an aqueous solution is well known to induce free radicals^9^. The cause is acoustic cavitation, a phenomenon whereby gas bubbles develop due to ebullism or evaporation and grow under negative pressure, and they then collapse under positive pressure as a result of pressure fluctuations caused by US. When bubbles collapse, they generate shock waves that induce localized high pressures of >600 atmospheres and temperature elevations of >5000 K. The energy created extends to neighboring water molecules, causing direct disintegration. This phenomenon (H_2_O→·OH +·H) is called sonolysis, and the ·OH, i.e. hydroxyl radical, is the most reactive of the various reactive oxygen species (ROS), including superoxide anion, singlet-dioxygen, and hydrogen peroxide. The production of ·OH by an ophthalmic phacoemulsificator was first reported by Cameron *et al*.^10^. They detected ·OH using electron spin resonance (ESR) in a closed circuit and demonstrated that ·OH production was proportional to the US oscillation time. We then also demonstrated ·OH production by ESR in the anterior chamber of a model eye under clinical conditions of phacoemulsification^11^. In addition, we proved that free radicals produced by phacoemulsification were the cause of corneal endothelial damage using 8-hydroxy-2-deoxyguanosine (8-OHdG) as an oxidative stress marker in animal eyes^12^.

To maintain the anterior chamber of the eye and to protect the corneal endothelium from various forms of surgical damage during phacoemulsification, an ophthalmic viscosurgical device (OVD) is injected into the anterior chamber before phacoemulsification as a standard procedure. The major ingredient of an OVD is sodium hyaluronate (HA), which is a known free radical scavenger. The protective properties of HA against oxidative stress in the corneal endothelium have been reported^13^, and we also demonstrated the scavenging effect of OVD against ·OH in a model eye study^11^,^14^. The effect of the material, however, depends on its retention in the anterior chamber during phacoemulsification^11^,^14^, because the material can be aspirated and disappear in the process of surgery. A new method of constant protection from oxidative insults during surgery has been awaited.

One promising candidate for this purpose is the hydrogen molecule, H_2_. In 2007, we reported that H_2_ selectively reduced cytotoxic ROS, ·OH, in particular, *in vitro* and exerted a therapeutic antioxidant activity in an ischemia-reperfusion injury model^15^. Since then, the effect of H_2_ against oxidative stress-induced injury in several organs, including the brain, heart, lung, intestine, or bone marrow, has been reported^16^,^17^,^18^,^19^. In the field of ophthalmology, we reported that H_2_ was effective to prevent ischemia reperfusion injury in the retina in a retinal artery occlusion model^20^. Considering the effect of H_2_ against ·OH, H_2_ dissolved in ocular irrigating solutions should work as a free radical scavenger in the anterior chamber. In this study, we examined the effect of H_2_ in phacoemulsification. We have demonstrated that US in phacoemulsification induces ·OH, which damages corneal endothelium^11^,^12^. If H_2_ dissolved in ocular irrigating solutions could prevent oxidative damage to the corneal endothelium, it would have great clinical usefulness.

## Results

### H_2_ concentration in the solution

**Figure 1:**
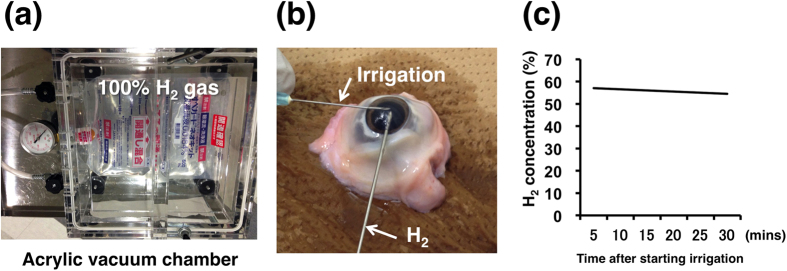
Preparation of H_2_-dissolved irrigating solution (Ope Guard^®^ neo kit) and H_2_ concentration measurement in the anterior chamber of a porcine eye. **(a)** Ope Guard^®^ neo kit is placed in an acrylic vacuum chamber, of which the air is replaced by 100% H_2_ gas. **(b)** After retrieval from the acrylic chamber, Ope Guard^®^ neo kit is immediately irrigated into the anterior chamber of an enucleated porcine eye at a speed of 10 ml/min. The H_2_ sensor is inserted through another incision site. **(c)** H_2_ concentration in the anterior chamber decreases gradually from 56.5% to 53.7% within 30 minutes.

To prepare the H_2_-dissolved ocular irrigating solution, bags containing the solution were placed in H_2_ gas. After 24-hour and 1-week exposure of the solution to 100% H_2_ gas in an acrylic chamber (Fig. 1a), the dissolved concentration of H_2_ in the solution was 61.9% and 61.8%, respectively, indicating that H_2_ gas easily penetrated through a plastic bag, and that the solution was almost equilibrated with H_2_ gas in the chamber within 24 hours. Thus, the H_2_-dissolved irrigating solution after 24-hour exposure was used for the following experiments. The H_2_ concentration in the anterior chamber of the porcine eye (Fig. 1b) under continuous irrigation (10 ml/min) with the H_2_-dissolved solution for 30 minutes showed little change, from 56.5% to 53.7% (Fig. 1c). To confirm O_2_ supply to the tissue, the O_2_ concentration was also measured. At 2 minutes of irrigation with the H_2_-dissolved solution, the O_2_ concentration in the anterior chamber was 5.4% ± 0.6%.

### Detection of ·OH by ESR and by HPF

**Figure 2:**
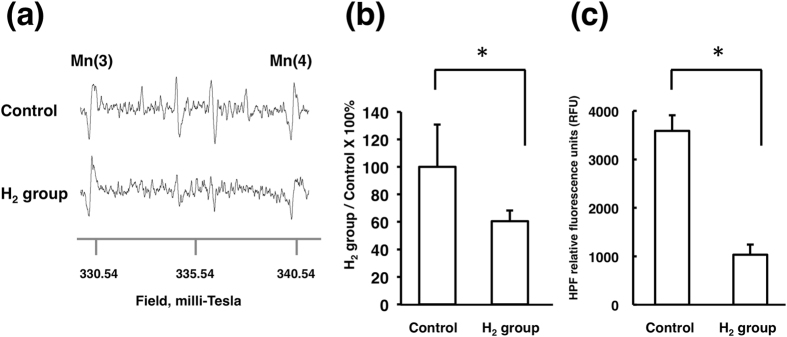
Detection of ·OH by ESR and by HPF. **(a)** Representative signals in the control and H_2_ groups on electron-spin resonance (ESR). Mn denotes the signal of manganese, which is used as a marker. The quartet signal suggesting DMPO-OH is obtained in both groups; however, the signal amplitude in the H_2_ group is apparently smaller than that of the control. **(b)** The intensities of the signals were calculated through image analysis after standardization using the amplitudes of the Mn signal. The signal intensity in the H_2_ group is significantly smaller than that of the control (each group n = 3, *P < 0.05 by the unpaired *t*-test). **(c)** HPF relative fluorescence units (RFU). RFU of the H_2_ group (1032.5 ± 324.7) is decreased to almost 30% of the control (3588 ± 210.0, *p < 0.05 by the unpaired *t*-test).

To demonstrate the reduction of ·OH with dissolved H_2_, ocular irrigating solutions containing DMPO were treated with the US device. Their ESR spin adducts showed the characteristic quartet signal pattern of DMPO-OH. The signal magnitude in the H_2_-dissolved solution was apparently suppressed (Fig. 2a). When the intensities of the signals were calculated by image analysis after standardization using the amplitudes of the Mn signal, the difference was significant (Fig. 2b; control 100 ± 30.8, H_2_ group 60.5 ± 7.8, *p* < 0.05). Similarly, ocular irrigating solutions containing HPF were treated with the US device. Their fluorescence intensity by oxidized HPF was significantly smaller in the H_2_-dissolved solution than in the control (Fig. 2c; Control 3588 ± 210.0, H_2_ group 1032.5 ± 324.7, *p* < 0.005).

### Image analysis of corneal edema

**Figure 3:**
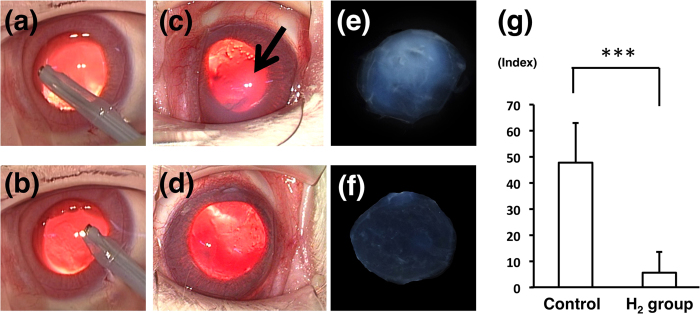
Photographs of the anterior segment and image analysis of corneal edema. **(a**,**b)** The position of the US probe, which was moved on the iris plane roundly without touching the lens in the control (**a**) and the H2 groups (**b**). **(c**,**d)** The anterior segment at 5 hours after surgery. The opaque lesion is apparent in the control eye (**c**; arrow), while in the H_2_ group, the opaque lesion is not clearly observed (**d**). **(e**,**f)** The representative image of the excised cornea of the control (**e**) and the H_2_ groups (**f**). **(g)** The intensity of the corneal opacity calculated using ImageJ software. The average index of the H_2_ group (n = 3, 5.6 ± 8) is significantly smaller than that of the control group (n = 4, 47.8 ± 15.2. ***p < 0.005 by the unpaired *t*-test).

Figure 3a (control) and b (H_2_ group) shows the US probe position in the anterior chamber of the rabbit when US oscillation was performed. The representative photographs of the anterior segment at 5 hours after US exposure are shown in Fig. 3c (control) and d (H_2_ group). The opaque, i.e. edematous lesion, was less apparent in the H_2_ group eye than in the control eye. To compare the amplitude of the edema quantitatively, the intensity of the opaque lesion was analyzed using ImageJ software. Representative images of the excised corneas in the control and the H_2_ groups are shown in Fig. 3e,f, respectively. The average index of the intensity was significantly smaller in the H_2_ group (5.6 ± 8) than in the control (47.8 ± 15.2) (Fig. 3g; *p* < 0.005).

### HO-1, CAT, SOD1, and SOD2 mRNA in corneal endothelial cells

**Figure 4:**
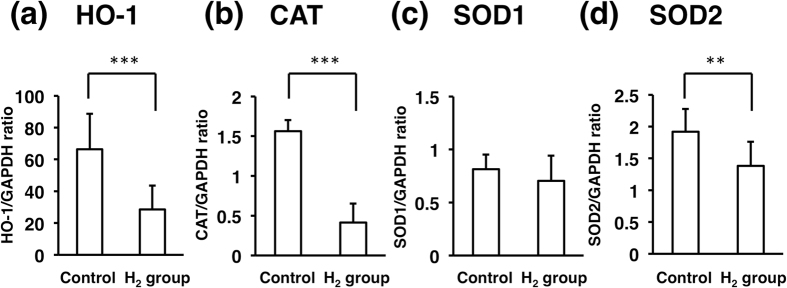
Expressions of HO-1, CAT, SOD1, and SOD2 mRNA in corneal endothelial cells. **(a)** HO-1 mRNA in corneal endothelial cells in the control (n = 7; 66.4 ± 22.3) and in the H_2_ group (n = 6; 28.5 ± 15). **(b)** CAT mRNA in corneal endothelial cells in the control (n = 7; 1.56 ± 0.42) and in the H_2_ group (n = 6; 0.42 ± 0.28). **(c)** SOD1 mRNA in corneal endothelial cells in the control (n = 7; 0.81 ± 0.14) and in the H_2_ group (n = 6; 0.7 ± 0.24). **(d)** SOD2 mRNA in corneal endothelial cells in the control (n = 7; 1.92 ± 0.36) and in the H_2_ group (n = 6; 1.38 ± 0.38). The difference between the 2 groups is significant (**p < 0.01 and ***p < 0.005 by the unpaired *t*-test).

At 5 hours after US exposure, the cornea was excised, and total RNA was extracted from the corneal endothelial cells. Real-time qPCR analysis of anti-oxidative heme oxygenase (HO)-1, catalase (CAT), superoxide dismutase 1 (SOD1), and SOD2 mRNAs showed that US exposure increased expressions of HO-1, CAT, and SOD2 in the corneal endothelium (Fig. 4a–d). The increase in HO-1 mRNA was significantly suppressed in the H_2_ group compared to the control (28.5 fold ± 15.0 vs. 66.4 fold ± 22.3, *p* < 0.005) (Fig. 4a). The increases in CAT and SOD2 mRNAs were also significantly suppressed in the H_2_ group compared to the control (0.42 fold ± 0.28 vs. 1.56 fold ± 0.88, *p* < 0.005 and 1.38 fold ± 0.38 vs. 1.92 fold ± 0.36, *p* < 0.01), respectively (Fig. 4b,d).

### Immunohistochemistry

**Figure 5:**
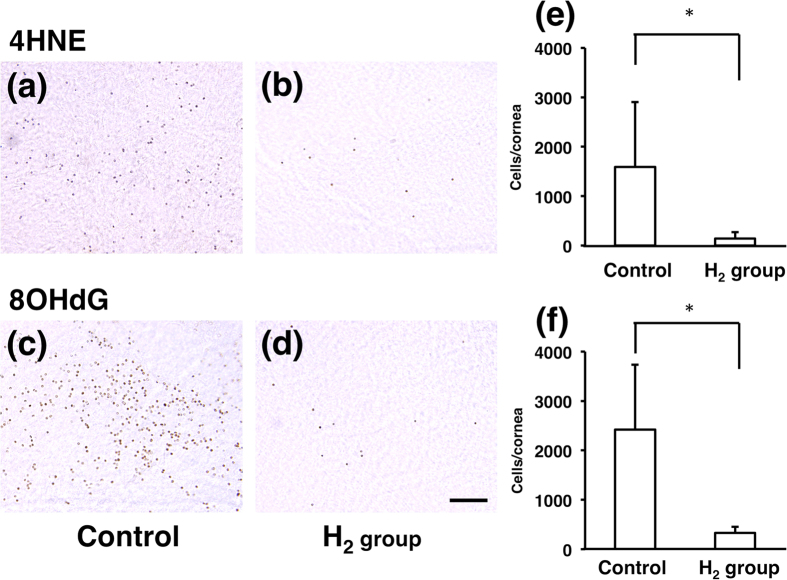
The oxidative stress marker in corneal endothelial cells. **(a–f)** Oxidative stress markers, 4-HNE (**a,b**) and 8-OHdG (**c,d**), in corneal endothelial cells at 5 hours after surgery. The 4-HNE-positive cells are significantly fewer in the H_2_ group (**b**; n = 5, 143.7 ± 124.6 cells/cornea) than in the control group (**a**; n = 3, 1586 ± 1315.6 cells/cornea) (**e**, *p < 0.05 by the unpaired *t*-test). The 8-OHdG-positive cells are significantly fewer in the H_2_ group (**d**; n = 6, 330.8 ± 730.9 cells/cornea) than in the control group (**c**; n = 5, 2518.7 ± 2294 cells/cornea) (**f**, *p < 0.05 by the unpaired *t*-test). Scale bar, 50 μm.

The expressions of two oxidative stress markers, 4-HNE and 8-OHdG, in the corneal endothelial cells at 5 hours after US exposure are shown in Fig. 5a–d, respectively. The number of 4-HNE-positive cells decreased in the H_2_ group (143.7 ± 124.6 cells/cornea) compared to the control (1586 ± 1315.6 cells/cornea, *p* < 0.05) (Fig. 5e). Furthermore, 8-OHdG-positive cells decreased in the H_2_ group (330.8 ± 730.9 cells/cornea) compared to the control (2518.7 ± 2294 cells/cornea, *p* < 0.05) (Fig. 5f).

## Discussion

Corneal endothelial damage induces corneal edema, and when the damage is excessive, it results in irreversible bullous keratopathy (BK). Because the corneal endothelial cells lack the ability to regenerate, transplantation of corneal tissue is the only cure currently available. A recent national survey on BK in Japan showed that cataract surgery was the most common cause of penetrating keratoplasty (24.2%)^21^, and a more recent study in the UK showed that, among the indications for endothelial keratoplasty, cataract surgery ranked second following Fuchs’ endothelial dystrophy^22^. Furthermore, corneal endothelial damage in a limited area, if it does not result in irreversible BK, is one of the most frequent complications after phacoemulsification and can cause temporary visual dysfunction in many post-phacoemulsification patients. Even though the safety of phacoemulsification has been dramatically improved due to progress in the apparatus and development of surgical devices including OVDs, the prevention of corneal endothelial damage in phacoemulsification is still an important issue for cataract surgeons.

The effect of H_2_ as a free radical scavenger has been vigorously investigated in various conditions *in vitro* or *in vivo*^23^, since our report first described its effect^15^. We first showed that H_2_ dose-dependently reduces ·OH *in vitro*, whereas H_2_ is too weak to reduce physiologically important ROS such as NO· and superoxide. H_2_, the smallest molecule in the universe, has the unique ability of rapidly diffusing across membranes; it can react with cytotoxic ·OH in all organelles, including mitochondria and the nucleus, and thus effectively protects cells against oxidative damage. Indeed, H_2_ prevented a decrease in the cellular levels of ATP synthesized in mitochondria^15^.

In phacoemulsification, ·OH production by US has been demonstrated^10^,^11^, and corneal endothelial damage by oxidative insults has also been demonstrated^12^. Considering the effect of H_2_ and the consequence of sonolysis in phacoemulsification, it seemed reasonable and worthwhile to investigate the usefulness of H_2_ dissolved in the irrigating solution, because, by this method, a potent free radical scavenger can be continuously provided during the surgery.

In the current study, the H_2_ concentration in the irrigating solution was examined first. H_2_ was dissolved into solution by placing the bag into a chamber of 100% H_2_. H_2_ spontaneously penetrates through the plastic film of the bag, even without positive pressure in the chamber. This was possible because the solution, Ope Guard^®^ neo kit, is a soft plastic product, which is why this solution was used. The H_2_ concentrations in the 24-hour and 1-week exposure solutions were almost the same, indicating H_2_ was almost saturated in the solution by 24-hour exposure with this method. Although this method has the advantage of eliminating concern about contamination, the H_2_ concentration in the bag decreased gradually after it was retrieved from the chamber, because H_2_ can escape from the bag. To confirm its validity in clinical use, H_2_ concentration was measured under clinical conditions. At 30 minutes of continuous irrigation, the H_2_ concentration in the anterior chamber was still 53.7%, suggesting that sufficient H_2_ concentration can be maintained during a standard phacoemulsification time (Fig. 1). Furthermore, the O_2_ concentration in the anterior chamber (5.4%) was confirmed to be within a safe range considering the short surgery time and the reported O_2_ concentration in the aqueous humor in the human eye^24^.

The effects of H_2_ in ·OH production caused by US were then examined in *in vitro* experiments using ESR and HPF analysis. US oscillation in the tube was performed as in our previous report^11^. Results of both analyses clearly demonstrated that H_2_ suppressed ·OH production (Fig. 2). Next, phacoemulsification simulation was performed in animal eyes to observe the effect of H_2_ in an *in vivo* model. In the procedure, the lens was not touched to avoid the effect of lens fragments on corneal endothelial damage. The difference between the H_2_ group and the control group was obvious. The area of the edematous lesion of the cornea evaluated by image analysis was significantly smaller in the H_2_ group (Fig. 3), suggesting that H_2_ protected the corneal endothelium from oxidative insults. To observe the physiological reaction to free radicals in the corneal endothelial cells, the expression of HO-1 mRNA was then examined in the cells after US exposure. HO-1 removes the pro-oxidant molecule heme and generates free radical scavengers, biliverdin and bilirubin; therefore, HO-1 is usually regarded as an antioxidant-inducible cellular defense protein, and an increase of HO-1 mRNA indicates that oxidative stress occurred in the cells^25^. The mRNA expressions of other anti-oxidative enzymes, including CAT, SOD1, and SOD2 were also examined, and all of them were suppressed in the H_2_ group, with significant changes in CAT and SOD2. Suppression of US exposure-induced HO-1, CAT, and SOD2 mRNA expressions in the H_2_ group (Fig. 4a,b,d) indicates that the corneal endothelial cells were protected by H_2_ from oxidative insults in phacoemulsification. Lastly, immunohistochemistry showed that the number of 4-HNE and 8-OHDG-positive cells was significantly smaller in the H_2_ group (Fig. 5), proving histologically that the oxidative damage was truly suppressed by H_2_.

All evidence presented in the current study strongly supports the usefulness of H_2_ in phacoemulsification. Among the various H_2_ applications in medicine reported so far, H_2_ dissolved in the ocular irrigating solution may be one of the most reasonable and promising uses of H_2_. In this system, H_2_ is continuously provided to the anterior chamber of the eye where ·OH is produced by US oscillation without any additional intervention such as injection or inhalation. The effect of H_2_ against ROS has been reported in various ways in previous studies^23^. Among these, although the effect in an ischemia-reperfusion injury mode has been regarded as the representative study since our first report^15^,^20^,^26^,^27^, we have also reported the effect of H_2_ in an irradiation-induced lung damage model^19^. One of the harmful effects of ionizing radiation, i.e. the indirect action, occurs when ionizing radiation interacts with water molecules in cells, which leads to production of ROS including ·OH. The ROS reacts rapidly with cellular macromolecules to damage DNA, lipids, and proteins and exert strong cytotoxic effects. It has been estimated that ·OH causes 60–70% of ionizing radiation-induced cell damage^28^. The sonolysis-induced ·OH in phacoemulsification is another mode of oxidative insult to the cells, and it seemed similar, at least partially, to irradiation-induced damage.

The standard composition of the ocular irrigating solution includes glutathione, which is a known anti-oxidative agent^29^,^30^. Although the solution, Ope Guard^®^ neo kit, used in the current study contains glutathione, production of ·OH shown by ESR and HPF was evident. However, the results of *in vivo* experiments clearly showed more corneal endothelial damage in the control than in the H_2_ group, suggesting that glutathione alone is not enough to protect cells from oxidative stresses caused by US oscillation in this model. In addition, several reports have shown the effect of ascorbic acid as an anti-oxidative agent in an *in vivo* phacoemulsification model^31^,^32^ and in an *in vitro* model^33^, but no clinical trial of ascorbic acid use in phacoemulsification has yet been done. This may be because ascorbic acid can be easily oxidized to dehydroascorbic acid, which can have an effect on glutathione^33^. In this regard, there is no concern about oxidative adducts with H_2_ use. Moreover, because there is literally no barrier to H_2_, it can function either outside or inside the cell membrane by rapid diffusion.

In conclusion, H_2_ dissolved in the ocular irrigating solution effectively protected corneal endothelial cells from oxidative stress and damage caused by phacoemulsification. H_2_ dissolved in the irrigating solution can have a dual action: as a direct scavenger against ·OH produced by sonolysis and as an anti-oxidative agent in the cell. In the current study, the former was clearly proven by the ESR and HPF analyses. In *in vivo* experiments, the favorable effect of H_2_ was evident, but to prove the anti-oxidative function of H_2_ in cells clearly, further investigation is needed.

## Methods

### Animals

Nine-week-old, male, Japanese white rabbits, weighing 2.5 to 3.0 kg, were purchased from Tokyo Laboratory Animals Science Co. Ltd. (Tokyo, Japan). The animals were kept individually under standardized laboratory conditions and given tap water and food *ad libitum*. All animals were treated in accordance with the ARVO Statement for the Use of Animals in Ophthalmic and Vision Research. Porcine eyes that were used for H_2_ concentration measurements were obtained from a local abattoir.

### Preparation of H_2_-dissolved irrigating solution

The ocular irrigating solution is used to maintain the anatomic and physiologic integrity of intraocular tissues in ocular surgeries including phacoemulsification. The standard composition of commercially available irrigating solutions includes glutathione, glucose, and sodium bicarbonate, which has been proven to be effective for maintenance of normal corneal endothelial functions^29^. In the current study, Ope Guard^®^ neo kit (Senju Pharmaceutical, Osaka, Japan), which contains the standard ingredients, was used. Ope Guard^®^ neo kit is a soft plastic bag product and one of the most popular irrigating solutions in Japan. To dissolve H_2_ in the solution, the bag was placed in an acrylic vacuum chamber (SNS-type, Sanplatec, Osaka, Japan), of which the air was replaced by 100% H_2_ gas (Fig. 1a). Because of its molecular size, H_2_ penetrates spontaneously through the plastic film of the bag even without positive pressure in the chamber. The bag was retrieved from the chamber after 24-hour or 1-week exposure, and the dissolved H_2_ concentration in the solution was immediately measured using a needle-type H_2_ sensor (Unisense, Aarhus N, Denmark). Then, to assess H_2_ concentration changes in the anterior chamber with irrigation of H_2_-dissolved solution, enucleated porcine eyes were used. The H_2_ sensor was inserted through the incision of an enucleated porcine eye, and H_2_ concentration was measured every 5 minutes for 30 minutes under continuous irrigation of the solution at 10 ml/min (Fig. 1b). Furthermore, to confirm O_2_ supply to the tissue, O_2_ concentration in the anterior chamber of an enucleated porcine eye at 2 minutes after the irrigation started was also measured using a needle-type O_2_ sensor (Unisense).

### Detection of ·OH by ESR

The ·OH detection by ESR was performed similarly to our previous paper^11^. For spin trapping, 10% aqueous solution of 5, 5′-dimethyl-1-pyrroline N-oxide (DMPO; Labotec, Tokyo, Japan) was used. DMPO was added to the Ope Guard^®^ neo kit solution at a final concentration of 1%. In 50-ml plastic test tubes, 10 ml of 1% DMPO/H_2_-dissolved solution (H_2_ group) or normal solution (control) was prepared. The US probe of a commercially available phacoemulsification device (Stellaris^®^, Bausch & Lomb, Rochester, NY) was placed in the center of the tube, and US was produced at a power level of 30% for 10 seconds without irrigation and aspiration. Immediately after US, 300 μl of the solution were transferred to a flat quartz ESR cuvette. The cuvette was then placed in an ESR spectrometer (model JES-RE3X; JEOL, Tokyo, Japan), and the signals of the spin adducts, DMPO-OH, were measured by double integration wave height using a computer software program (ES-IPEITS data system version 7.0; JEOL). All measurements were performed 3 times.

### Detection of ·OH by HPF

HPF is a novel reagent that directly detects certain highly reactive oxygen species (hROS)^34^. Although HPF itself has little fluorescence, it selectively and dose-dependently reacts with hROS, such as ·OH and peroxynitrite, and shows strong fluorescence. HPF (Goryo Chemical, Hokkaido, Japan) was added to H_2_-dissolved solution (H_2_ group) or normal solution (control) at a final concentration of 5 μM. As in the ESR experiment, in 10 ml of the solution in the 50-ml plastic test tubes, US oscillation without irrigation and aspiration was performed for 10 seconds at 30% power. Immediately after US, fluorescence of the samples was measured by a plate reader (Wallac 1420 ARVO; PerkinElmer, Waltham, MA) with excitation at 485 nm and emission at 535 nm. All measurements were performed 4 times.

### Effects of H_2_ in phacoemulsification *in vivo*

Rabbits were anesthetized via intramuscular injection of ketamine (30 mg/kg) and xylazine (4 mg/kg), and topical oxybuprocaine hydrochloride (Santen Pharmaceutical, Osaka, Japan) was used for local anesthesia. The US probe (Stellaris^®^, Bausch & Lomb) was introduced into the center of the anterior chamber through the incision, and then continuous US oscillation with irrigation by H_2_-dissolved solution (H_2_ group) or normal solution (control) was performed for 90 seconds at 30% power of the US setting of the device (vacuum pressure: 175 mmHg; bottle height: 85 cm). To avoid the effect of probe manipulation, the phacoemulsification procedure was performed in a completely similar fashion in both groups. The US probe was moved slowly on the iris plane roundly without touching the lens. At 5 hours after the procedure, the rabbits were euthanized by overdose injection of ketamine. After the anterior segments were photographed using the ophthalmologic surgical microscope, the whole cornea was excised and submitted to the following experiments.

### Image analysis of corneal edema

The excised corneas (control n = 4, H_2_ group n = 3) were photographed and analyzed using ImageJ software (Version 1.44, NIH, Bethesda, MD) as described previously^35^,^36^. Each image was captured using the same camera settings for gain and time, and pixel intensity was analyzed for 25,000 pixels (500 × 500 pixels) at the center of the cornea. Background intensity was calculated from the untreated cornea and subtracted from each image.

### Semiquantitative PCR for HO-1, CAT, SOD1, and SOD2 mRNA

HO-1 has been known to play an important role in cell membrane protection against various oxidative stresses^25^. CAT, SOD1, and SOD2 are also crucial for breaking down the harmful end products of oxidative phosphorylation^37^. The effect of H_2_ on their mRNA expression in the corneal endothelium was evaluated. At 5 hours after US exposure, the cornea was excised, and the endothelial cells were immediately scraped using a blunt scalpel. Total RNA was extracted from corneal endothelial cells and subjected to reverse transcription, with portions of the resulting cDNA then subjected to real-time semi-quantitative PCR (qPCR) analysis with a 7500 Fast Real-Time PCR System (Life Technologies, Tokyo, Japan). The Fast Real-Time PCR System was used with the following primers: for HO-1, 5′-CAGGTGACTGCCGAGGGTTTTA-3′ (forward) and 5′-GGAAGTAGAGCGGGGCGTAG-3′ (reverse)^38^; for CAT, 5′-CCCAATAGGAGACAAACTGA-3′ (forward) and 5′-ACTCTCTCCGGAATTCTCTC-3′ (reverse) were designed using DNASIS Pro software (Hitachi Software Engineering Co., Ltd., Tokyo, Japan); for SOD1, 5′-GACGCATAACAGGACTGACCG-3′ (forward) and 5′-AACACATCAGCGACACCATTG-3′ (reverse)^39^, for SOD2, 5′-TGACGGCTGTGTCTGTTGGT-3′ (forward), and 5′-GCAGGTAGTAAGCGTGTTCCC-3′ (reverse)^39^, for rabbit glyceraldehyde -3-phosphate dehydrogenase (GAPDH) gene, 5′-GCCGCTTCTTCTCGTGCAG-3′ (forward) and 5′-ATGGATCATTGATGGCGACAACAT-3′ (reverse) (accession no. L23961)^40^. The cycling protocol entailed incubation at 90 °C for 10 s followed by 40 cycles of 95 °C for 5 s and 60 °C for 34 s. Relative gene expression was calculated using the standard curve method. The HO-1 mRNA levels were normalized to those of the housekeeping GAPDH gene. The HO-1/GAPDH ratio in untreated corneal endothelial cells was defined as 1.0. To assess the average value of the HO-1/GAPDH ratio, measurements were performed 5 times. Each sample was run in duplicate, and each real-time PCR was repeated three times (control n = 7, H_2_ group n = 6).

### Immunohistochemistry

The excised corneas were fixed in Bouin’s solution (Wako Pure Chemical Industries, Osaka, Japan) for 1 hour at 4 °C, washed 3 times for 10 minutes each in PBS with 1% Triton X-100 (Bio-Rad Laboratories, CA) (PBST), and treated with acetone for 3 minutes at −20 °C. They were further washed 3 times for 10 minutes with PBST and incubated in 10% horse serum diluted in PBST to block nonspecific staining. The corneas were then incubated in a 1:30 dilution of a monoclonal antibody against 8-OHdG (control n = 5, H_2_ group n = 6) or 4-hydroxy-2-nonenal (4-HNE) (control n = 3, H_2_ group n = 5) overnight at 4 °C (18). Both antibodies were purchased from the Japan Institute for the Control of Aging (Shizuoka, Japan). The corneas were washed with PBST 3 times for 10 minutes and incubated with horse biotinylated anti-mouse IgG (Vector Laboratories, Burlingame, CA) for 1 hour at room temperature. Tissues were then washed with PBST 3 times for 10 minutes and incubated with ABC reagent (Vector Laboratories) for 1 hour at room temperature. After being washed with PBST 3 times for 10 minutes, corneas were stained with DAB solution (Vector Laboratories) for 10 minutes at room temperature. Finally, they were washed with distilled water for 5 minutes each and mounted on slides.

### Statistics

Morphometric data from different regions in each eye were averaged to provide one value per eye. The mean and SD for these measurements were calculated for each group, and comparisons between groups were made using the unpaired *t*-test (Stat Flex ver. 6, Artec, Osaka, Japan). A *p* value of <0.05 was considered significant.

### Study approval

This experiment was approved by the Animal Care and Use Committee of Nippon Medical School (27–045).

## Additional Information

**How to cite this article**: Igarashi, T. *et al*. Hydrogen prevents corneal endothelial damage in phacoemulsification cataract surgery. *Sci. Rep*. **6**, 31190; doi: 10.1038/srep31190 (2016).
